# Inflammatory bowel disease-associated ubiquitin ligase RNF183 promotes lysosomal degradation of DR5 and TRAIL-induced caspase activation

**DOI:** 10.1038/s41598-019-56748-6

**Published:** 2019-12-30

**Authors:** Yan Wu, Yuka Kimura, Takumi Okamoto, Koji Matsuhisa, Rie Asada, Atsushi Saito, Fumika Sakaue, Kazunori Imaizumi, Masayuki Kaneko

**Affiliations:** 1 Department of Biochemistry, Graduate school of Biomedical and Health Sciences, Hiroshima University, Hiroshima, Japan; 2 Department of Medicine, Division of Endocrinology, Metabolism, and Lipid Research, Washington University School of Medicine, MO St. Louis, USA; 3 Department of Stress Protein Processing, Graduate School of Biomedical and Health Sciences, Hiroshima University, Hiroshima, Japan

**Keywords:** Ubiquitylated proteins, Ubiquitin ligases, Ubiquitylation, Ubiquitin ligases, Ubiquitylation, Ubiquitylation, Ubiquitin ligases, Ubiquitylation, Ubiquitylation

## Abstract

RNF183 is a ubiquitin ligase containing RING-finger and transmembrane domains, and its expression levels are increased in patients with inflammatory bowel disease (IBD), including Crohn’s disease and ulcerative colitis, and in 2,4,6-trinitrobenzene sulfonic acid-induced colitis mice. Here, we further demonstrate that RNF183 was induced to a greater degree in the dextran sulfate sodium (DSS)-treated IBD model at a very early stage than were inflammatory cytokines. In addition, fluorescence-activated cell sorting and polymerase chain reaction analysis revealed that RNF183 was specifically expressed in epithelial cells of DSS-treated mice, which suggested that increased levels of RNF183 do not result from the accumulation of immune cells. Furthermore, we identified death receptor 5 (DR5), a member of tumour necrosis factor (TNF)-receptor superfamily, as a substrate of RNF183. RNF183 mediated K63-linked ubiquitination and lysosomal degradation of DR5. DR5 promotes TNF-related apoptosis inducing ligand (TRAIL)-induced apoptosis signal through interaction with caspase-8. Inhibition of RNF183 expression was found to suppress TRAIL-induced activation of caspase-8 and caspase-3. Thus, RNF183 promoted not only DR5 transport to lysosomes but also TRAIL-induced caspase activation and apoptosis. Together, our results provide new insights into potential roles of RNF183 in DR5-mediated caspase activation in IBD pathogenesis.

## Introduction

Inflammatory bowel disease (IBD) is a group of inflammatory conditions of the colon and small intestine, including Crohn’s disease (CD) and ulcerative colitis (UC)^1^. IBD results from chronic dysregulation of the mucosal immune system in the gastrointestinal tract. However, the molecular mechanisms underlying the development and pathophysiology of IBD are not fully understood. Recent study has revealed that expression levels of RING-finger protein 183 (RNF183), which functions as a ubiquitin ligase and predominantly localises to lysosomes^2^, in the colon of patients with IBD were 5-fold higher than those in control subjects; in these patients, RNF183 promoted intestinal inflammation^3^.

Ubiquitination is mediated by ubiquitin ligase (E3) and repeated to form polyubiquitin chains. Ubiquitin itself contains seven lysine residues (Lys6, Lys11, Lys27, Lys29, Lys33, Lys48, and Lys63) and the initiator methionine that can serve as acceptor sites for chain elongation^4,5^. Ubiquitination has multiple roles not only in proteasome-mediated protein degradation but also in the targeting of membrane proteins for degradation inside the lysosome. Ubiquitination provides key signals to membrane proteins for endocytosis and endosomal sorting into the multivesicular body, which delivers its cargo to the proteolytic interior of the lysosome^6,7^. There are > 600 putative ubiquitin ligases in the human genome^8^; however, many have been poorly characterized, particularly their protein substrates.

IBD models can be induced in mice by dextran sulphate sodium (DSS) in the drinking water or by a 2,4,6-trinitrobenzene sulfonic acid (TNBS)-ethanol enema, which evoke immune responses and colitis^9,10^. In this study, we investigated DSS-induced RNF183 expression in mice colons. In DSS colitis mice, compared with inflammatory cytokines, RNF183 was expressed at a very early stage and specifically in epithelial cells. Furthermore, we identified death receptor 5 (DR5) as a substrate of RNF183. DR5, also called tumour necrosis factor (TNF) receptor superfamily member 10B (TNFRSF10B) and TNF-related apoptosis inducing ligand (TRAIL; also called TNFSF10 and APO-2L) receptor 2 (TRAILR2), is a cell surface receptor of the TNF-receptor superfamily^11^. This receptor contains an intracellular death domain and transduces apoptosis signalling through interaction with caspase-8^12,13^. A previous study showed that DR5 is decreased in the large intestine epithelial tissue of patients with CD and UC^14^. Furthermore, DR5 knockout mice are more susceptible to DSS-induced colitis^15^. However, the underlying molecular mechanisms of DR5 in IBD remain unclear. Here, we demonstrated that RNF183 induced K63-linked ubiquitination-mediated lysosomal degradation of DR5 and caspase activation.

## Result

### RNF183 expression increased in a DSS-induced colitis mouse model

**Figure 1:**
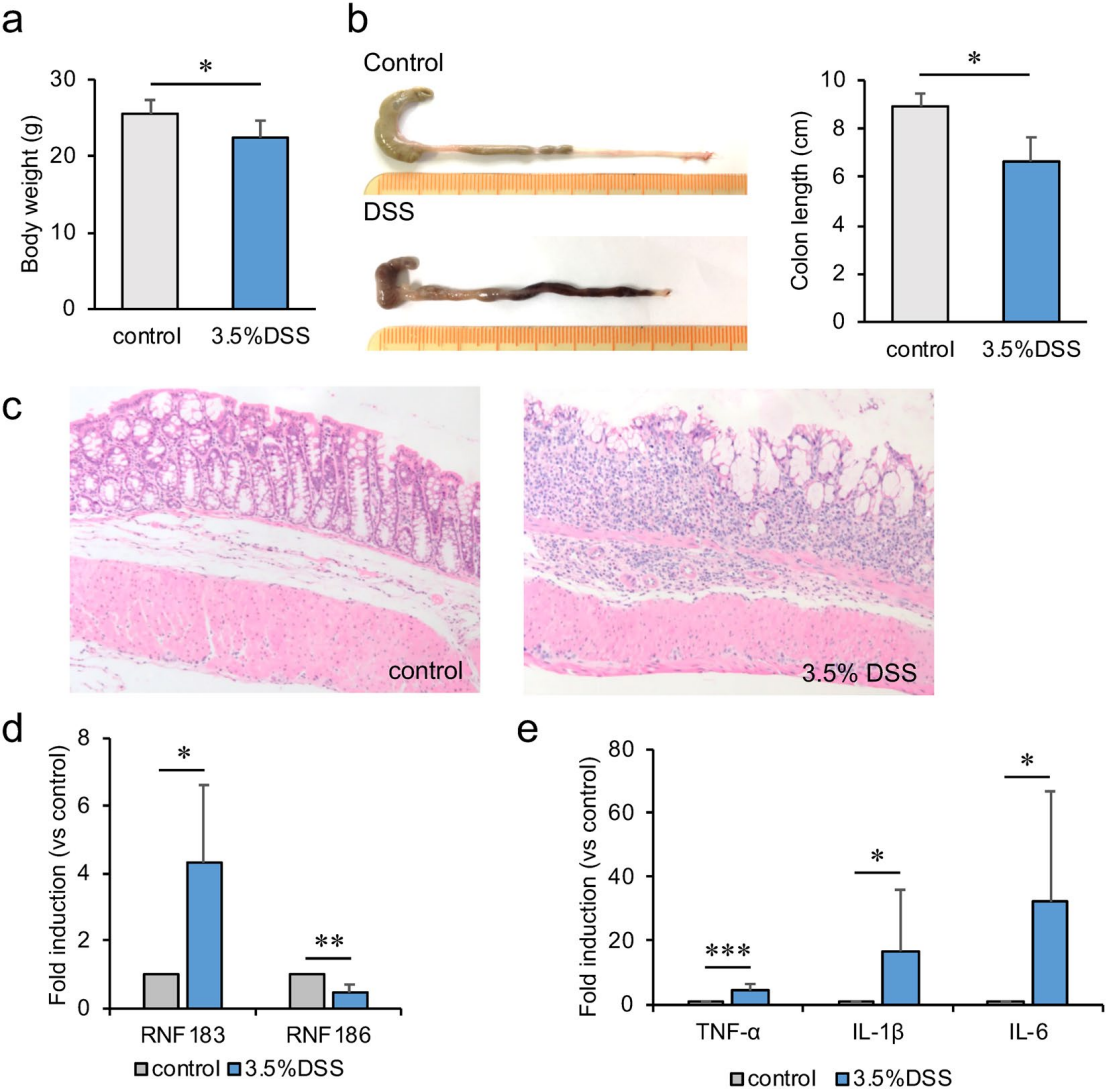
RNF183 expression in the dextran sulfate sodium (DSS)-induced colitis mouse model. (**a**) Changes in body weight and (**b**) colon length between the control (water) and DSS-treated mice after 5 days exposure. Asterisks represent significant differences (n = 6; Student’s *t*-test, *p < 0.05). (**c**) Haematoxylin-eosin (HE) staining of mouse colon tissue exposed to control (water) and 3.5% DSS for 5 days ( × 20 magnification). (**d**) Expression of RNF183 and RNF186 mRNA in whole colon tissues between control and 3.5% DSS-treated mice at 5 days. (**e**) The mRNA levels of inflammatory markers in whole colon tissues between control and 3.5% DSS-treated mice at 5 days. The expression levels were determined by quantitative reverse transcription polymerase chain reaction (qRT-PCR) Asterisks represent significant differences (n = 6; paired *t*-test, *p < 0.05, **p < 0.01, ***p < 0.001).

RNF183 mRNA and protein have been reported to be highly expressed in inflamed colon tissue of patients with UC and CD^3^. RNF183 expression has also been induced in the mouse colon of TNBS colitis model^3^. Furthermore, RNF186, a gene closely related to RNF183, was identified as a disease-susceptibility gene for UC from genome-wide association studies^16^. Thus, we examined whether RNF183 and RNF186 expressions are increased in another IBD model. An acute colitis model was established by 3.5% DSS in the drinking water for 5 days. Significant reduction in body weight (Fig. 1a) and shortening in colon length (Fig. 1b) were observed in DSS-treated mice after 5 days of exposure. Concomitantly, the colon tissue of the DSS-treated mice showed loss of crypts and goblet cells and mucus layer and substantial neutrophil infiltration into the lamina propria (Fig. 1c), which indicated successful establishment of the IBD model with DSS. The mRNA levels of RNF183 and inflammatory markers were compared between water controls and DSS-treated mice by using quantitative reverse transcription polymerase chain reaction (qRT-PCR). RNF183 mRNA level was significantly increased in DSS-treated mice compared with that in control mice, whereas RNF186 was not increased (Fig. 1d). TNF-α, IL-1β, and IL-6 mRNA levels were markedly upregulated in the DSS-treated mice group (Fig. 1e).

### RNF183 mRNA expression increased at an early stage of DSS-induced colitis

**Figure 2:**
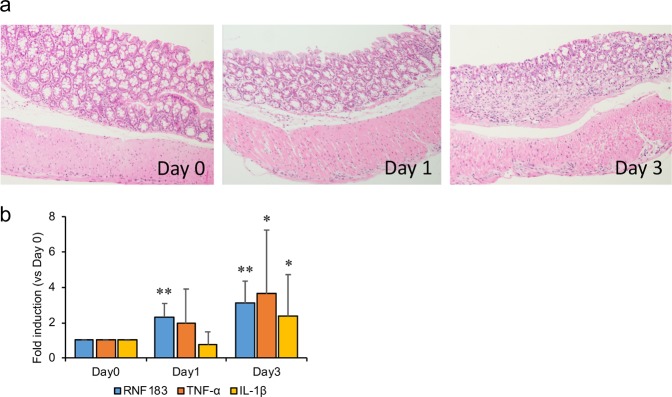
RNF183 expression at the early stage of DSS-induced colitis. (**a**) HE staining of colon tissues of DSS-treated mice at 0, 1, and 3 days ( × 20 magnification). (**b**) The expressions of RNF183, TNF-α, and IL-1β mRNA in DSS-treated mice at 0, 1, and 3 days. The expression levels were determined by qRT-PCR. Asterisks represent significant differences (n = 5; one-way analysis of variance (ANOVA), followed by Dunnett’s test, *p < 0.05, **p < 0.01; 0 day vs. 1 and 3 days).

Our results indicate the possibility that DSS-induced RNF183 expression is caused by a secondary inflammatory response. To determine when RNF183 is expressed in DSS-induced colitis, we investigated RNF183 expression at an early stage of DSS-induced colitis. To compare the progression of pathological conditions and expression of genes, the disease activity index (DAI) was used to assess DSS colitis severity, as performed in previous studies^17,18^. Health status was investigated at 1, 3, and 5 days, including weight of the animal, stool consistency, and presence of rectal bleeding (Table 1). DSS-treated mice at 5 days had the highest clinical scores. These assessed parameters gradually increased with increasing duration of DSS treatment. The colonic tissue of the DSS-treated mice at 1 and 3 days was observed by haematoxylin-eosin (HE) staining (Fig. 2a). DSS-treated mice at 3 days showed modest inflammatory infiltration, whereas those at 1 day did not show any change. We examined RNF183, TNF-α, and IL-1β mRNA levels after 1 and 3 days of DSS exposure by using qRT-PCR. RNF183 mRNA expression was significantly elevated at 1 day, whereas TNF-α and IL-1β mRNA levels were not significantly changed at 1 day (Fig. 2b). This quick response of RNF183 without signs of significant inflammatory responses suggests that DSS-induced RNF183 expression does not result from a secondary inflammatory response caused by increased inflammatory cytokines.

**Table 1:** Disease activity index (DAI) for assessment.

DSS dosing day	Weight loss		Stool consistency		Rectal bleeding		Score
Day 1	1–3%	1	well-formed pellets	0	normal	0	**1**
Day 3	4–10%	2	pasty	1	hemorrhage	2	**5**
Day 5	10–20%	3	liquid stools	2	gross bleeding	4	**9**

Development of DSS-induced inflammation was daily assessed on the basis of the changes in the following parameters over time: weight of the animal, stool consistency, and presence of rectal bleeding. Final score is the sum of the assessed parameters.

### RNF183 was expressed in colonic epithelial cells of DSS-treated mice

**Figure 3:**
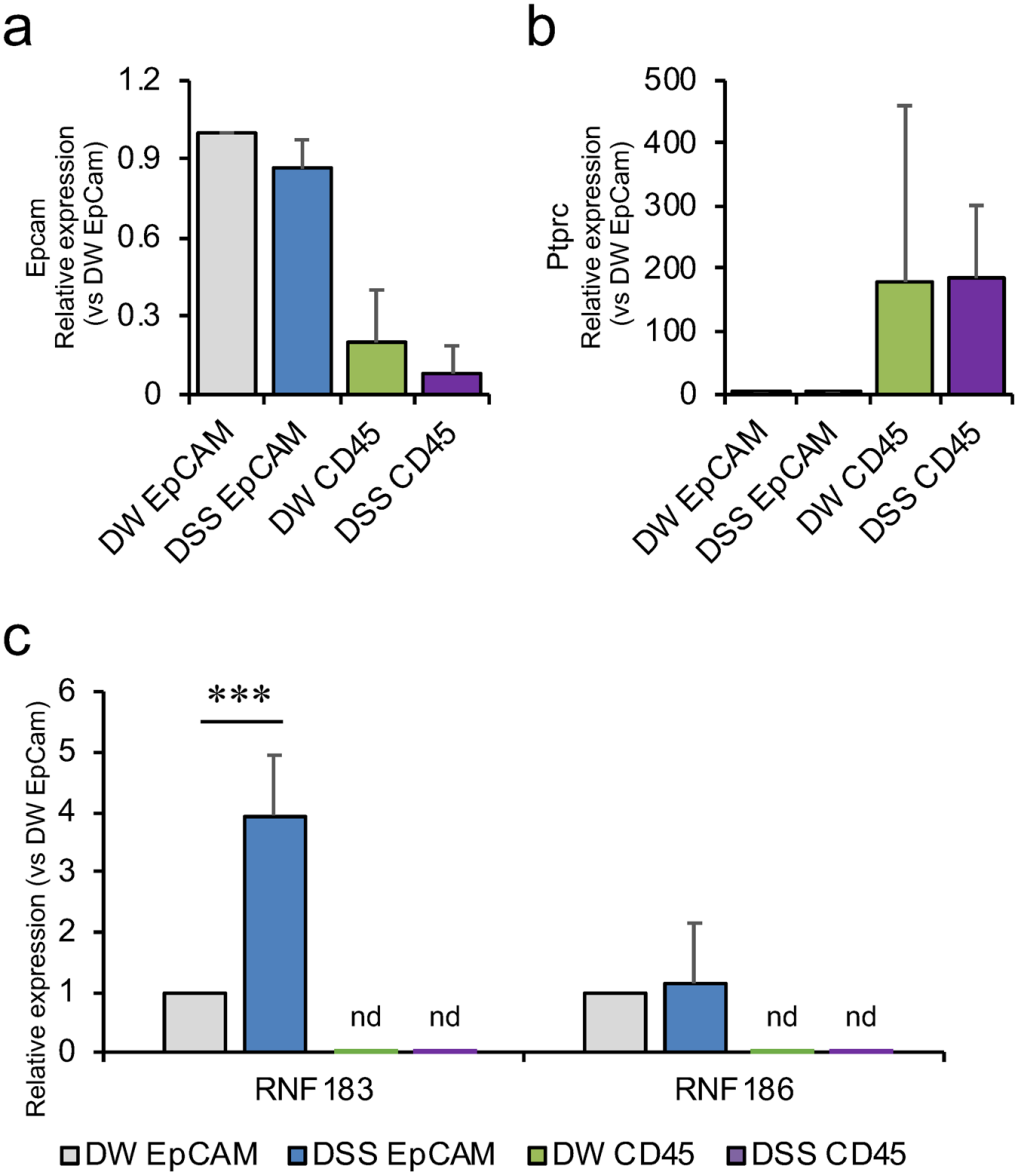
RNF183 expression in colonic epithelial cells of DSS-treated mice. (**a**) Epcam (CD326) and (**b**) Ptprc (CD45) mRNA levels in isolated colonic epithelial and immune cells, respectively. Isolated colonic cells from drinking water (DW)- and DSS-treated mice at 5 days were labelled with anti-CD326 (PE) and anti-CD45 (FITC) antibodies and sorted by fluorescence-activated cell sorting. The expression levels in sorted cells were determined by qRT-PCR. (**c**) RNF183 and RNF186 mRNA levels in colonic epithelial and immune cells from DW- and DSS-treated mice at 5 days. The expression levels in sorted cells were determined by qRT-PCR. Asterisks represent significant differences (n = 5; paired *t*-test, ***p < 0.001). nd: not detected.

It remains unclear where DSS-induced RNF183 localises because there is no specific anti-RNF183 antibody. To determine which colonic cells express RNF183, we isolated epithelial and immune cells from colon tissues of the DSS-treated mice by using fluorescence-activated cell sorting. CD326 (Epithelial cell adhesion molecule, EpCAM) and CD45 (receptor-type tyrosine-protein phosphatase C, PTPRC) fluorescent antibodies were used for sorting of epithelial cells (Fig. 3a) and immune cells (Fig. 3b), respectively; we checked their corresponding gene expression by using qRT-PCR. qRT-PCR analysis of sorted cells revealed that the RNF183 mRNA levels in DSS-treated CD326-positive epithelial cells were significantly increased relative to those in CD326-positive non-treated control cells, consistent with the experimental result on the whole colon tissues (Fig. 3c). However, RNF183 in CD45-positive cells was not detected in both DSS-treated and non-treated control cells (Fig. 3c), although CD45-positive immune cells were successfully isolated from colon tissues (Fig. 3b). Thus, these results indicate that RNF183 was selectively expressed in colonic epithelial cells, but not immune cells.

### Identification of DR5 as a substrate of RNF183

**Figure 4:**
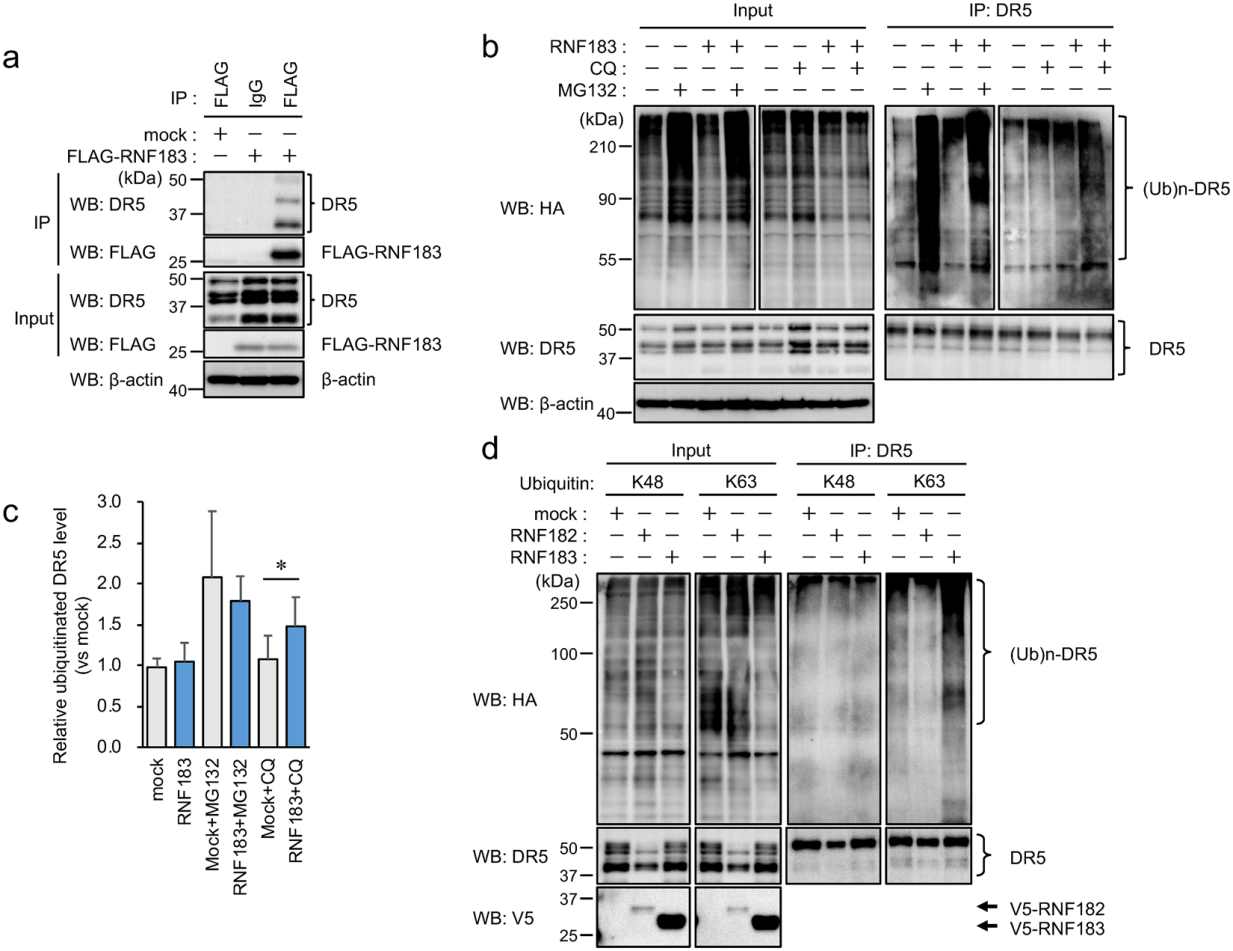
Interaction of DR5 with RNF183 and its ubiquitination. (**a**) Co-immunoprecipitation of RNF183 and DR5. Cell lysates from HEK293 cells stably expressing FLAG-tagged RNF183 were immunoprecipitated with anti-FLAG antibody, and the immune complexes were analysed by western blotting with anti-DR5 or anti-FLAG antibodies. IP; immunoprecipitation. The full-length blots are presented in Supplementary Fig. S7. (**b**) Ubiquitination of DR5 by RNF183. HEK293 cells stably expressing V5-tagged RNF183 were transfected with HA-tagged ubiquitin. At 36 h after transfection, the cells were incubated with or without 10 μM MG132 or 100 μM chloroquine (CQ) for 12 h. Cell lysates were immunoprecipitated with anti-DR5 antibody, and the immune complexes were analysed by western blotting with anti-HA or anti-DR5 antibodies. The full-length blots are presented in Supplementary Fig. S8. (**c**) Quantitative graph of data from (**b**). Asterisks represent significant differences (n = 5; paired *t*-test, *p < 0.05). (**d**) K48- and K63-linked ubiquitination of DR5. HEK293 cells stably expressing V5-tagged RNF183 or RNF182 or mock-transfected cells were transfected with HA-tagged ubiquitin K48 or K63 mutants. Cell lysates were immunoprecipitated with anti-DR5 antibody. The full-length blots are presented in Supplementary Fig. S9.

To elucidate the role of RNF183 in IBD, we identified RNF183-interacting proteins. We performed a mass spectrometric analysis of proteins that were co-immunoprecipitated with RNF183 from human embryonic kidney HEK293 cells engineered to stably express RNF183. DR5 was identified as a candidate for RNF183 substrate (number of detected peptides, 1; peptide cover rate, 3.86%). DR5 is a cell surface receptor of the TNF-receptor superfamily that binds TRAIL and activates caspase-8. Next, we confirmed the interaction between RNF183 and DR5 by co-immunoprecipitation. Since RNF183 expression is normally restricted to the renal collecting duct^19^, there is no cell line expressing RNF183 under isotonic conditions^20^. Therefore, in the present study, we mainly used HEK293 cells stably expressing physiological levels of RNF183. Exogenously expressed RNF183 interacted with endogenous DR5 (Fig. 4a). Furthermore, we determined if RNF183 ubiquitinates DR5. HA-tagged-ubiquitin was transfected into HEK293 cells stably expressing RNF183 in the presence of proteasome inhibitor MG132 or lysosome inhibitor chloroquine. Ubiquitin chains immunoprecipitated with DR5 were significantly increased by RNF183 in the presence of chloroquine, whereas the ubiquitin chains were not increased by RNF183 in MG132 treatment relative to those in non-treated controls (Fig. 4b,c). Ubiquitin-dependent lysosomal degradation is mediated by K63-linked polyubiquitination. To determine whether RNF183-mediated DR5 ubiquitination uses K63- or K48-ubiquitin chains, we used two mutant ubiquitins that can form only K63 or K48 chains with other lysines mutated to arginines. As opposed to K48, K63 ubiquitin can form polyubiquitin chains on DR5 in the presence of RNF183 (Fig. 4d). On the other hand, lysosomal ubiquitin ligase RNF182, which is a closely related gene to RNF183, was unable to ubiquitinate DR5^19^ (Fig. 4d). In addition, we used the Tandem Ubiquitin Binding Entity (TUBE) system to isolate K63-linked poly-ubiquitinated proteins from HEK293 expressing wild-type (WT) or point mutations (CS) of RNF183 in which consensus cysteines of the RING finger domain are substituted by serine, induced by the Tet-On system. TUBE1 (K63-linked poly-ubiquitin-specific binding beads) pull-down followed by DR5 western blot assay demonstrated that poly-ubiquitinated DR5 was significantly increased in WT-RNF183 cells compared with those of CS mutant and uninduced cells. Moreover, poly-ubiquitinated DR5 was increased by chloroquine treatment (Supplementary Fig. S1). These results indicate that RNF183 mediates K63-linked poly-ubiquitin chains of DR5.

### RNF183 degraded DR5 by its transport to lysosomes

**Figure 5:**
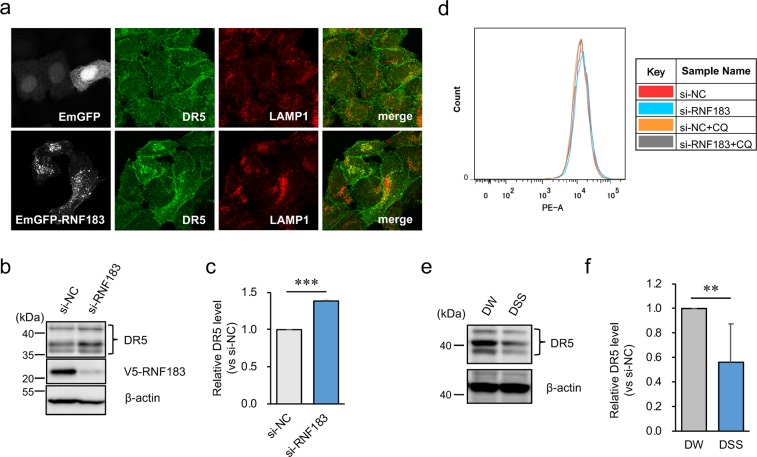
Involvement of RNF183 in the localization and degradation of DR5. (**a**) Effect of RNF183 overexpression on DR5 subcellular localization. HeLa cells transfected with EmGFP-tagged RNF183 or EmGFP alone were subjected to immunofluorescence staining with DR5 and LAMP1 antibodies (*grey*, EmGFP; *green*, DR5; *red*, LAMP1). (**b**) Effect of RNF183 knockdown on DR5 protein levels. RNF183-siRNA or non-target control (NC)-siRNA were transfected into HEK293 cells stably expressing V5-tagged RNF183. Cell lysates were subjected to western blotting with DR5 and V5 antibodies. The full-length blots are presented in Supplementary Fig. S10. (**c**) Quantitative graph of data from (**b**). Asterisks represent significant differences (n = 3; paired *t*-test, ***p < 0.001). (**d**) Cell surface expression level of DR5. HEK293 cells expressing V5-tagged RNF183 were treated with 100 μM CQ for 12 h. Then, cells were labeled with PE-conjugated anti-DR5 antibody. (**e**) DR5 protein levels in colonic epithelial cells from DW- and DSS-treated mice at 5 days. Cell lysates were subjected to western blotting with anti-DR5 and anti-β-actin antibodies. The full-length blots are presented in Supplementary Fig. S11. (**f**) Quantitative graph of data from (**e**). Asterisks represent significant differences (n = 6; paired *t*-test, **p < 0.01).

Since ubiquitinated DR5 was accumulated by RNF183 in the presence of chloroquine, we determined if DR5 is transported by RNF183 to lysosomes by immunofluorescence staining. HeLa cells were transfected with emerald green fluorescent protein (EmGFP)-tagged RNF183 and labelled with DR5 and the lysosome marker LAMP1. Only a small part of DR5 was localised to lysosomes when cells were transfected with EmGFP alone as a control (Fig. 5a, upper panels). In contrast, DR5 was translocated into the intracellular space and largely localised to lysosomes when cells were transfected with EmGFP-tagged RNF183 (Fig. 5a, lower panels). In the z-axial direction, DR5 still did not colocalize with LAMP1 in the absence of RNF183 (Supplementary Fig. S2a), whereas DR5 and LAMP1 signals were merged in the intracellular space in the presence of RNF183 (Supplementary Fig. S2b). Chloroquine treatment showed increased colocalization of DR5 and LAMP1, indicating lysosomal accumulation, either with or without RNF183 (Supplementary Fig. S2c). Moreover, the deleted RING (Δ R), which lacks the RING-finger domain, CS mutations of RNF183, and RNF182 exhibited the same pattern as control (Supplementary Fig. S3).

Next, to determine if DR5 degradation is promoted by RNF183, RNF183-siRNA was transfected into HEK293 cells stably expressing RNF183. As expected, when RNF183 expression was inhibited by siRNA, the DR5 protein level significantly increased relative to that in the non-target control (NC)-siRNA (Fig. 5b,c). In contrast, the DR5 protein level in RNF183-expressed cells was lower than that in mock-transfected cells (Supplementary Fig. S2d). In addition, DR5 protein levels were also increased by chloroquine treatment, consistent with confocal images (Supplementary Fig. S2d).

Here, it raises the question as to where RNF183 ubiquitinates DR5. To address this question, DR5 cell surface expression levels were determined by flow cytometry analysis. DR5 cell surface expression levels were not significantly changed with or without RNF183 expression in the presence or absence of chloroquine (Fig. 5d), suggesting that RNF183 is not involved in DR5 endocytosis. Taken together, these results suggest that RNF183 promotes the trafficking of DR5 from endosomes to lysosomes and degradation of DR5 in the lysosome.

To support the link between the increase in RNF183 expression and DR5 protein degradation, we determined DR5 protein levels in isolated colonic epithelial cells from the colons of DSS-treated mice at 5 days. As expected, the DR5 expression was decreased in the DSS-treated mice (Fig. 5e,f). On the other hand, DR5 mRNA levels were not significantly changed between controls and DSS-treated mice (Supplementary Fig. S4). These results imply that RNF183 could promote DR5 protein degradation in DSS-treated mice.

### RNF183 promoted TRAIL-induced apoptosis

**Figure 6:**
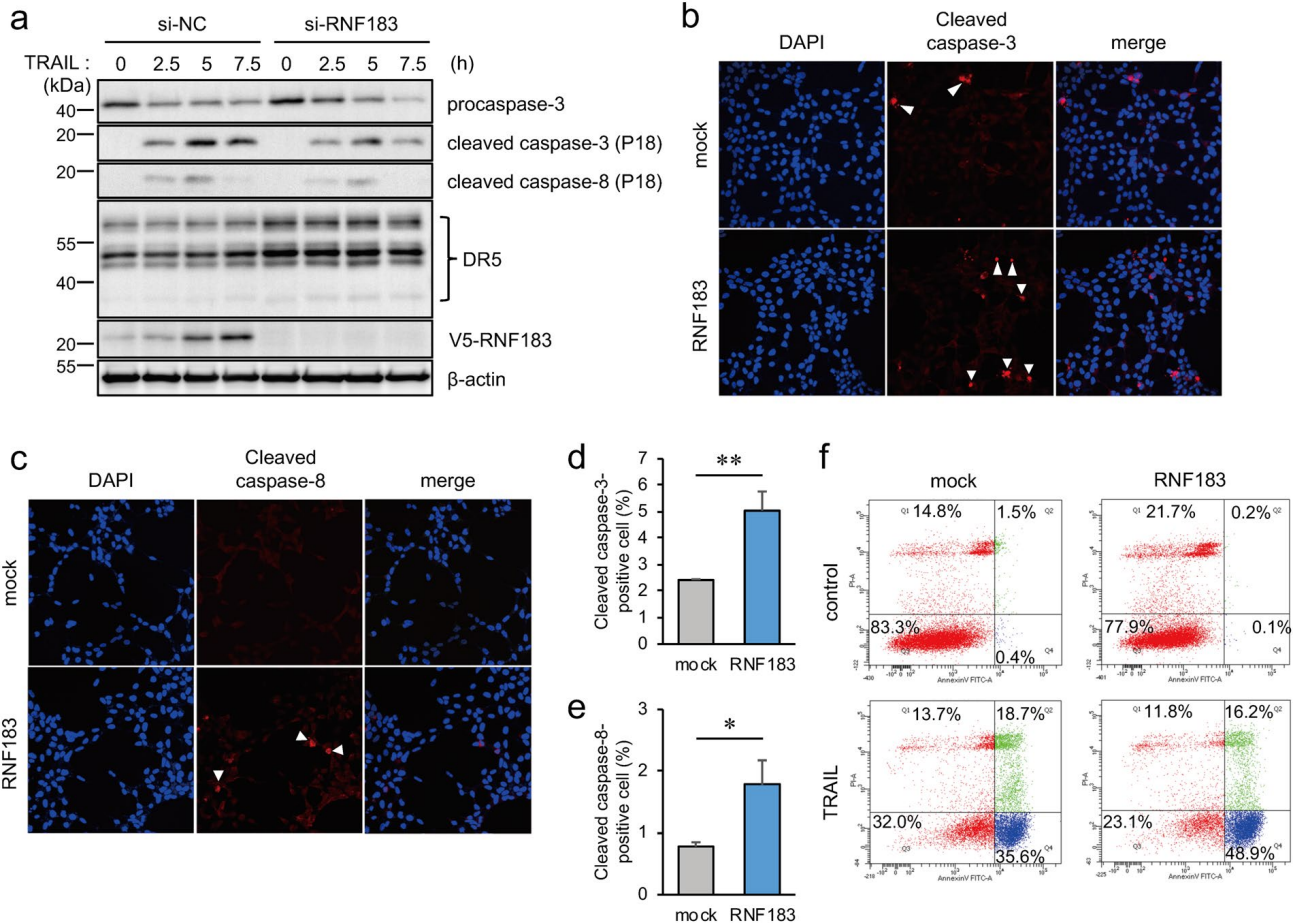
Involvement of RNF183 in TRAIL-induced apoptosis. (**a**) Effect of RNF183 knockdown on TRAIL-induced caspase activation. RNF183-siRNA was transfected into HEK293 cells stably expressing V5-tagged RNF183. At 30 h after transfection, TRAIL (100 ng/mL) was added and cells were incubated for 2.5, 5, and 7.5 h. Cell lysates were subjected to western blotting with anti-cleaved caspase-3, cleaved caspase-8 antibodies, and anti-caspase-3 antibody (for procaspase-3). The full-length blots are presented in Supplementary Fig. S12. (**b**,**c**) Effect of RNF183 overexpression on TRAIL-induced caspase activation. HEK293 cells expressing mock or V5-tagged RNF183 were treated with TRAIL for 5 h. Cells were subjected to immunofluorescence staining with cleaved caspase-3, cleaved caspase-8 antibodies (*red*), and DAPI (*blue*). (**d**,**e**) Quantitative graph of data from (**b**,**c**). Asterisks represent significant differences (n = 3; Student’s *t*-test, *p < 0.05, **p < 0.01). (**f**) Apoptotic cell death was evaluated by Annexin V assay. HEK293 cells expressing mock or RNF183 were treated with TRAIL (300 ng/ml) for 3 h. The percentage of apoptotic cells was determined by FITC-annexin-V/propidium iodide (PI) staining.

TRAIL binds to DR5 that can activate caspase-8, resulting in caspase-3 activation and apoptosis. Thus, we determined if RNF183 is involved in TRAIL-induced apoptosis. RNF183-siRNA was transfected into HEK293 cells stably expressing RNF183 in the presence of TRAIL. The expressions of cleaved caspase-3 and caspase-8 were decreased by RNF183 knockdown relative to those in NC-siRNA transfected cells (Fig. 6a). Moreover, HEK293 cells stably expressing RNF183 in the presence of TRAIL were subjected to immunofluorescence staining with cleaved caspase-3 and caspase-8 antibodies. RNF183-expressed cells exhibited a significant increase in both cleaved caspase-3–and caspase-8–positive cells relative to the increase in mock-transfected cells (Fig. 6b–e). In contrast, knockdown of RNF183 in HEK293 cells stably expressing RNF183 exhibited a decreased activation of caspase-3 and caspase-8 relative to NC-siRNA controls (Supplementary Fig. S5). Additionally, TRAIL-induced apoptotic cell death of HEK293 cells stably expressing mock or RNF183 cells was evaluated by flow cytometry using Annexin V. Early apoptosis cells were apparently increased in RNF183-expressing cells (mock: 35.6%; RNF183: 48.9%) (Fig. 6f).

Because some reports have described that death receptor 4 (DR4) rather than DR5 dominantly triggers apoptosis upon TRAIL stimulation in a number of cancer cell lines^21^, we also evaluated the association of RNF183 with DR4. We examined the interaction between RNF183 and DR4 by co-immunoprecipitation. In contrast to DR5, DR4 did not interact with RNF183 (Supplementary Fig. S6a). In addition, when RNF183 expression was inhibited by siRNA, the DR4 protein level was not significantly changed compared with that of controls (Supplementary Fig. S6b, c). These results suggest that RNF183 is not associated with DR4 protein degradation. Therefore, RNF183 may regulate TRAIL-induced apoptosis through DR5 ubiquitination.

## Discussion

In our previous study, we found that RNF183 was specifically expressed in the kidney under normal conditions^2^. Although RNF183 expression is extremely low in the colon, a recent study demonstrated that RNF183 expression was strongly induced in inflamed colon samples from patients with IBD and TNBS-induced colitis mice^3^. Here, we investigated whether RNF183 expression is induced by another IBD model mouse by using DSS and determined when and where RNF183 is expressed in a DSS colitis model. Intriguingly, our results revealed that DSS-induced RNF183 expression rapidly increased relative to increases in proinflammatory cytokines, such as IL-1β, TNF-α, and IL-6. There are no other genes similar to RNF183 that are induced in the acute DSS colitis model at an early stage (1 day), whereas most of the cytokine and chemokine genes were expressed at a later stage (2 and 4 days)^18^. Another RNF family gene, RNF186, was significantly decreased in the acute DSS colitis model. RNF186 variants in patients with UC have also been identified^16^. In addition, RNF186 plays an important role in maintaining homeostasis of the colon through occluding ubiquitination^22^.

On the other hand, our cell sorting and PCR analyses demonstrated that RNF183 was specifically expressed in colonic epithelial cells, but not immune cells, during DSS-induced colitis. Combined with the observation that immune cell infiltration was not observed after a 1-day exposure to DSS, it is likely that increased levels of RNF183 at 1 day does not result from the accumulation of immune cells. Therefore, RNF183 expressed at such an early stage of DSS-induced colitis may have an important role in the pathogenesis of IBD. In fact, the involvement of RNF183 in inflammation has been reported to occur in the following ways: 1) RNF183 induced NF-κB activation through degrading IκBα, which contributes to the pathogenesis of IBD^3^. 2) RNF183 promoted proliferation and metastasis of colorectal cancer cells via activation of the NF-κB−IL-8 axis^23^. On the other hand, we found that RNF183 was upregulated by hypertonic stress through the hypertonicity-responsive transcription factor NFAT5 in the collecting duct^20,24^. Notably, patients with IBD are substantially exposed to higher osmolality of the faeces compared with control subjects (~490 vs. ~340 mOsmol/kg H_2_O)^25,26^. In addition, NFAT5 is abundantly expressed in colonic epithelial cells, consistent with RNF183 expression^27^. Thus, disruption of the mucosal barrier probably induces RNF183 expression via NFAT5 in colonic epithelial cells. Therefore, experiments in conditional NFAT5-knockout mice should reveal the involvement of NFAT5 in DSS-induced RNF183 expression and RNF183-mediated pathogenesis of IBD.

We identified DR5 as a substrate of RNF183 by co-immunoprecipitation coupled with mass spectrometry. In the present study, we focused on DR5 because DR5 was reported to be involved in IBD and DSS-mediated colitis^14,15,28^. We previously found that RNF183 is predominantly localised in lysosomes^2^. Furthermore, knockdown of Sec. 16 A, a key protein in COPII vesicle formation, has been shown to inhibit RNF183 export from the endoplasmic reticulum (ER) and accelerate its rapid degradation, suggesting that RNF183 functions downstream of the ER^2^. Consistent with this finding, RNF183 overexpression enhanced lysosomal localization of DR5; accumulation of ubiquitinated DR5 was caused by chloroquine but not MG132 in the presence of RNF183. Therefore, DR5 degradation mediated by RNF183-induced ubiquitination likely occurs in the lysosome, but not the ER (so-called ER-associated degradation). Mono-ubiquitination or K63-linked poly-ubiquitination of cell surface membrane proteins, such as receptor- and transporter induce endocytosis and lysosomal targeting. The ubiquitin ligase c-Casitas B-lineage lymphoma (c-Cbl) mediates mono-ubiquitination of DR5, which leads to lysosomal degradation^29–31^. We demonstrated for the first time that RNF183 can promote K63-linked poly-ubiquitination of DR5 and subsequent lysosomal degradation. Consistent with our results from the DSS-treated mice, immunohistochemical analysis revealed that DR5 was selectively downregulated in the surface epithelium of IBD patients^14^. Therefore, RNF183-mediated DR5 downregulation could occur in patients with IBD.

Although RNF183 knockdown caused DR5 protein accumulation, TRAIL-induced apoptosis was reduced by RNF183 knockdown. This discrepancy suggests that DR5 ubiquitination by RNF183 promotes TRAIL-induced caspase activation and DR5 transport to lysosomes. TNFRSF, including DR5, has been shown to be internalized by endocytosis and to activate downstream pathways from endosomal compartments^32^. However, the requirement of DR5 endocytosis for caspase-8 activation has been controversial because it is both indispensable^33^ and dispensable^34^. Flow cytometric analysis revealed that RNF183 did not affect DR5 cell surface expression levels, suggesting that RNF183-mediated ubiquitination does not promote DR5 endocytosis. Some previous studies have demonstrated that TRAIL-induced internalization of DR5 leads to transport to lysosomes, resulting in lysosomal protease release into the cytosol to promote apoptosis^33,35–37^. Therefore, it is likely that internalized DR5 can be activated by RNF183 in endosomes or lysosomes before its degradation. On the other hand, our results demonstrated that caspase-3 and caspase-8 were activated by RNF183, which indicated that RNF183 could induce apoptosis through caspase-8–mediated caspase-3 activation. Apoptosis of epithelial cells was increased in intestinal tissues from patients with UC and CD^38,39^. However, in IBD, caspase-8 activation protects intestinal epithelial cells through necroptosis rather than apoptosis^40,41^. These findings indicate the possibility that RNF183 could protect intestinal epithelium tissue of IBD through activation of caspase-8. Thus, to determine if upregulated RNF183 has protective or deleterious effects on epithelial cells in IBD, experiments in RNF183 knockout mice should be performed.

In summary, we showed that ubiquitin ligase RNF183 expression was increased in colonic epithelial cells of DSS-induced IBD model mouse at a very early stage. RNF183 promoted poly-ubiquitination of DR5 and transport to lysosomes, which eventually led to caspase-8–mediated apoptosis. Considering these findings, RNF183 may be associated with the pathogenesis of IBD and considered to be a potential novel drug target for IBD if its involvement in colitis is demonstrated in an IBD model mouse.

## Materials and Methods

### Antibodies

Antibodies against the following proteins were used in this research: β-actin (AC-15, mouse monoclonal; A5441, Sigma-Aldrich, St. Louis, MO); CD326 (EpCAM) PE (G8.8, mouse monoclonal; 12-5791-81, Thermo Fisher Scientific, Waltham, MA); CD45 FITC (30-F11, mouse monoclonal; 11-0451-81, Thermo Fisher Scientific); caspase-3: (rabbit polyclonal; #9662, Cell Signaling Technology, Danvers, MA): cleaved caspase-3: (D175, rabbit monoclonal; #9661, Cell Signaling Technology); cleaved caspase-8: (18C8, rabbit monoclonal; #9496, Cell Signaling Technology); DR5 (D4E9, rabbit monoclonal; #8074, Cell Signaling Technology); FLAG Tag (M2, mouse monoclonal; F1084, Sigma-Aldrich); goat anti-mouse IgG H&L (Alexa Fluor 647) pre-adsorbed (ab150119, Abcam, Cambridge, UK); goat anti-rabbit IgG (H + L) cross-adsorbed secondary antibody, Alexa Fluor 568 (A-11011, Thermo Fisher Scientific); HA Tag (6E2, mouse monoclonal; #2367, Cell Signaling Technology); LAMP1 (H4A3, mouse monoclonal; sc-20011, Santa Cruz Biotechnology, Dallas, TX); normal rat lgG2a, PE (sc-2872, Santa Cruz Biotechnology); rat IgG2b kappa isotype control (eB149/10H5), FITC (11-4031-81, Thermo Fisher Scientific); V5 Tag (mouse monoclonal; R960-25, Thermo Fisher Scientific).

### Expression vectors and stable cell lines

We followed previously established protocols for preparation of the expression vectors and stable cell lines^2,19^.

### Mice and dextran sulphate sodium (DSS)-induced acute colitis

C57BL/6 J mice (ages 8–10 weeks old, weight 22–27 g, male) were obtained from Hiroshima experimental animals. All experiments were conducted in accordance with the US National Institutes of Health Guidelines for the Care and Use of Laboratory Animals. The experimental procedures and housing conditions for the animals were approved by the Committee of Animal Experimentation, Hiroshima University. The mice were divided into two groups (n = 6). The Control group was provided sterilized drinking water; the DSS group was provided sterilized drinking water with 3.5% DSS MW 36,000−50,000 (MP Biomedicals, Solon, OH) for 5 days. The mice were checked each day for morbidity, and weight was recorded after administration of 3.5% DSS. Afterwards, the mice were sacrificed by cervical dislocation.

### Cell culture

Human embryonic kidney HEK293 cells and human cervix HeLa cells were maintained in Dulbecco’s Modified Eagle’s Medium (Thermo Fisher Scientific) supplemented with 10% (v/v) heat-inactivated foetal bovine serum (FBS) at 37 °C in a 5% CO_2_, 95% humidified air atmosphere.

### Histological analysis

The distal large intestines of adult mice were fixed overnight in 10% formalin (FUJIFILM Wako Pure Chemical, Osaka, Japan). Then, the samples were dehydrated with ethanol, embedded in paraffin, and sectioned at 5–6 µm. HE staining was performed according to standard protocols. HE-stained colon sections were pathologically scored as described previously^42^.

### Disease activity index (DAI) for assessment

DAI for assessment of acute colitis treated with 3.5% DSS for 1, 3, and 5 days. The scoring criteria were based on a previous study^17,18^.

### Isolation of colonic epithelial cell

Preparation of colonic epithelial cells from the large intestines of adult mice was performed according to previously published protocols^43,44^ with some modifications. Initially, after the colon was removed and washed with phosphate-buffered saline (PBS), the colon was washed with solution A (96 mM NaCl, 27 mM sodium citrate, 1.5 mM KCl, 0.8 mM KH_2_PO_4_, and 5.6 mM Na_2_HPO_4_•12H_2_O in sterilized water, pH 7.4). Rectangular pieces of tissue were placed in 10 ml of solution A at 37 °C for 10 min with gentle shaking. The tissue fragments were then incubated in 3 ml of solution B (0.1 mM EDTA, 115 mM NaCl, 25 mM NaHCO_3_, 2.4 mM K_2_HPO_4_, 0.4 mM KH_2_PO_4_, and 2.5 mM glutamine in sterilized water, pH 7.4) at 37 °C for 30 min with gentle shaking. Digestion was stopped by addition of 100 mM CaCl_2_. The total tissue fragments in liquid were then vortex mixed for 10 s. The liquid was centrifuged at 300 × g at 4 °C. The precipitate was collected and used as colonic epithelial cells.

### Flow cytometry and sorting

For isolation of epithelial cells, isolated colonic epithelial cells were blocked with Fc Block [CD16/32 (mouse BD Fc Block; Becton, Dickinson, and Company, Franklin Lakes, NJ) in 1% bovine serum albumin (BSA)/PBS] on ice for 10 min, and then centrifuged for 5 min at 300 × g at 4 °C. Cells were labelled with 1 µg of anti-CD326 (PE) and anti-CD45 (fluorescein isothiocyanate) antibodies, or normal IgG as control in the dark for 20 min at 4 °C, and the cells were washed with 3 ml of 1% BSA/PBS 3 times. A 600-µl aliquot of 2% FBS/1 mM EDTA/PBS was added, and the cells were gently resuspended. The cells were filtered through a 100-μm pore size filter, to prevent clogging. A FACSAria II (Becton, Dickinson and Company) was used to isolate the epithelial (CD326) and immune (CD45) cells.

For cell surface expression levels of DR5, the cells were harvested by trypsinization and washed with ice cold PBS containing 10% FBS and 1% sodium azide. Approximately 2 × 10^6^ cells in 100 μl of PBS with 3% BSA were incubated for 30 min in the dark with PE-conjugated anti-DR5 antibody (catalog no. 307406, BioLegend, San Diego, CA, USA). PE-labeled isotype IgG1 antibody (catalog no. 400112, BioLegend) was used as a negative control. The cells were filtered through a 30 μm pore size filter to prevent clogging. LSRFortessa X-20 (Becton, Dickinson and Company) was used to analyze the cell surface expression levels of DR5.

For the annexin V assay, the MEBCYTO Apoptosis Kit (Annexin V-FITC Kit) was used according to the manufacturer’s instructions (MEDICAL & BIOLOGICAL LABORATORIES, Nagoya, Japan). First, approximately 10^6^ cells were seeded on a 3.5 cm plate; the next day, apoptosis was induced by TRAIL (300 ng/ml) for 3 h. Cells were treated with trypsin before being harvested and then gently washed once with PBS. After staining with Annexin V-FITC and propidium iodide and filtration through a 30 μm pore size filter, the cell samples were measured by LSRFortessa X-20.

### Quantitative reverse transcription polymerase chain reaction (qRT-PCR)

Total RNA was isolated from the large intestine and colonic epithelial cells of mice treated with 3.5% DSS by using ISOGEN (NIPPON GENE, Tokyo Japan) according to the manufacturer’s protocol. An equal amount (100–200 ng) of total RNA was used for reverse transcription cDNA synthesis using SuperScript IV VILO Master Mix with ezDNase (Thermo Fisher Scientific). The reverse-transcribed cDNA was measured by TaqMan based real-time PCR assay using a LightCycler 480 Instrument II (Roche Diagnostics, Basel, Switzerland). The following primer and probe sets, purchased from Integrated DNA Technologies (Coralville, IA), were used: Epcam: Mm.PT.58.11851150; Il1b: Mm.PT.58.41616450; Ptprc: Mm.PT.58.7583849; Rnf183: Mm.PT.58.43079040; Rnf186: Mm.PT.56a.29338718.g; Tnf: Mm.PT.58.12575861; Tnfrsf10b: Mm.PT.58.32192376.

### Immunocytochemistry

HEK293 cells were transiently transfected with EmGFP-tagged RNF183 using ScreenFect A (FUJIFILM Wako Pure Chemical) and grown on poly-L-lysine coated coverslips. After 30 h, the cells were fixed with 4% paraformaldehyde for 15 min and blocked with 5% normal goat serum in 0.3% Triton X-100/PBS at room temperature for 60 min. The cells were then stained with anti-DR5 and anti-LAMP1 antibodies in 1% BSA/0.3% Triton X-100/PBS overnight at 4 °C. Subsequently, the cells were incubated with Alexa Fluor 568-conjugated goat anti-rabbit IgG and Alexa Fluor 647-conjugated goat anti-mouse IgG secondary antibodies for 60 min at room temperature. A ProLong Diamond Antifade Mountant with DAPI (Thermo Fisher Scientific) was used to mount coverslips on the slides. Fluorescence images were acquired by using a FLUOVIEW FV1000 (Olympus Corporation, Tokyo, Japan).

### Immunoprecipitation and western blotting

The interaction of RNF183 and DR5: HEK293 cells stably expressing FLAG-tagged RNF183 were lysed in lysis buffer [20 mM Tris-HCl (pH 7.5), 150 mM NaCl, 10% glycerol, 1% Triton X-100, 100 μM MG132 (FUJIFILM Wako Pure Chemical), Protease inhibitor cocktail Set V (EDTA free; FUJIFILM Wako Pure Chemical) on ice for 20 min. Supernatants were incubated with an anti-FLAG antibody at 4 °C for 1 h, and followed by incubation with Protein G Agarose Beads (Thermo Fisher Scientific) for 1 h; subsequently, the beads were rinsed three times with a wash buffer [20 mM Tris-HCl (pH 7.5), 150 mM NaCl, 10% glycerol, 0.1% Triton X-100]. Immunoprecipitates were boiled with Laemmli sodium dodecyl sulphate polyacrylamide gel electrophoresis sample buffer and analysed by western blotting.

The ubiquitination of DR5:HEK293 cells stably expressing V5-tagged RNF183 were lysed in lysis buffer containing 20 µM MG132, 1 mM N-ethylmaleimide (FUJIFILM Wako Pure Chemical), 5 mM EDTA on ice for 20 min, and then quenched with 0.1% cysteine. Supernatants were incubated with anti-DR5 antibody at 4 °C for 1 h, followed by incubation with Protein G Agarose Beads for 1 h; the beads were rinsed with the same wash buffer.

### Proteome analysis

Shotgun proteomic analysis was performed as described previously^2^. FLAG-tagged RNF183 and mock precipitates obtained as described in the previous section were used as the proteome analysis sample.

### Statistical analysis

All data are expressed as the mean ± standard deviation. Comparisons between two groups were analysed using the two-tailed Student’s *t*-test or paired *t*-test. For multiple-group comparisons, one-way analysis of variance (ANOVA), followed by Dunnett’s test.

## Supplementary information

Supplementary information.

## References

[1] Podolsky DK (2002). Inflammatory bowel disease. N. Engl. J. Med..

[2] Wu Y (2018). Sec. 16A, a key protein in COPII vesicle formation, regulates the stability and localization of the novel ubiquitin ligase RNF183. PLoS One.

[3] Yu Q (2016). E3 Ubiquitin ligase RNF183 Is a Novel Regulator in Inflammatory Bowel Disease. J. Crohns Colitis.

[4] Pickart CM (2001). Mechanisms underlying ubiquitination. Annu. Rev. Biochem..

[5] Grabbe C, Husnjak K, Dikic I (2011). The spatial and temporal organization of ubiquitin networks. Nat. Rev. Mol. Cell Biol..

[6] Haglund K, Dikic I (2012). The role of ubiquitylation in receptor endocytosis and endosomal sorting. J. Cell Sci..

[7] Piper R. C., Dikic I., Lukacs G. L. (2014). Ubiquitin-Dependent Sorting in Endocytosis. Cold Spring Harbor Perspectives in Biology.

[8] Li W (2008). Genome-wide and functional annotation of human E3 ubiquitin ligases identifies MULAN, a mitochondrial E3 that regulates the organelle’s dynamics and signaling. PLoS One.

[9] Okayasu I (1990). A novel method in the induction of reliable experimental acute and chronic ulcerative colitis in mice. Gastroenterology.

[10] Wirtz S (2017). Chemically induced mouse models of acute and chronic intestinal inflammation. Nat. Protoc..

[11] Screaton GR (1997). TRICK2, a new alternatively spliced receptor that transduces the cytotoxic signal from TRAIL. Curr. Biol..

[12] Hymowitz SG (1999). Triggering cell death: the crystal structure of Apo2L/TRAIL in a complex with death receptor 5. Mol. Cell.

[13] Mongkolsapaya J (1999). Structure of the TRAIL-DR5 complex reveals mechanisms conferring specificity in apoptotic initiation. Nat. Struct. Biol..

[14] Brost S (2010). Differential expression of the TRAIL/TRAIL-receptor system in patients with inflammatory bowel disease. Pathol. Res. Pract..

[15] Zhu J (2014). TRAIL receptor deficiency sensitizes mice to dextran sodium sulphate-induced colitis and colitis-associated carcinogenesis. Immunology.

[16] Beaudoin M (2013). Deep resequencing of GWAS loci identifies rare variants in CARD9, IL23R and RNF186 that are associated with ulcerative colitis. PLoS Genet..

[17] Kaser A (2008). XBP1 links ER stress to intestinal inflammation and confers genetic risk for human inflammatory bowel disease. Cell.

[18] Pushparaj PN (2013). Interleukin-33 exacerbates acute colitis via interleukin-4 in mice. Immunology.

[19] Kaneko M (2016). Genome-wide identification and gene expression profiling of ubiquitin ligases for endoplasmic reticulum protein degradation. Sci. Rep..

[20] Maeoka Y (2019). NFAT5 up-regulates expression of the kidney-specific ubiquitin ligase gene Rnf183 under hypertonic conditions in inner-medullary collecting duct cells. J. Biol. Chem..

[21] Dufour F (2017). TRAIL receptor gene editing unveils TRAIL-R1 as a master player of apoptosis induced by TRAIL and ER stress. Oncotarget.

[22] Fujimoto K (2017). Regulation of intestinal homeostasis by the ulcerative colitis-associated gene RNF186. Mucosal Immunol..

[23] Geng R (2017). RNF183 promotes proliferation and metastasis of colorectal cancer cells via activation of NF-kappaB-IL-8 axis. Cell Death Dis..

[24] Maeoka Y (2019). Renal medullary tonicity regulates RNF183 expression in the collecting ducts via NFAT5. Biochem. Biophys. Res. Commun..

[25] Schilli R (1982). Comparison of the composition of faecal fluid in Crohn’s disease and ulcerative colitis. Gut.

[26] Vernia P, Gnaedinger A, Hauck W, Breuer RI (1988). Organic anions and the diarrhea of inflammatory bowel disease. Dig. Dis. Sci..

[27] Jauliac S (2002). The role of NFAT transcription factors in integrin-mediated carcinoma invasion. Nat. Cell Biol..

[28] Begue B (2006). Implication of TNF-related apoptosis-inducing ligand in inflammatory intestinal epithelial lesions. Gastroenterology.

[29] Woo SM, Kwon TK (2019). E3 ubiquitin ligases and deubiquitinases as modulators of TRAIL-mediated extrinsic apoptotic signaling pathway. BMB Rep..

[30] Song JJ (2010). c-Cbl-mediated degradation of TRAIL receptors is responsible for the development of the early phase of TRAIL resistance. Cell Signal..

[31] Kim SY, Kim JH, Song JJ (2013). c-Cbl shRNA-expressing adenovirus sensitizes TRAIL-induced apoptosis in prostate cancer DU-145 through increases of DR4/5. Cancer Gene Ther..

[32] Cendrowski J, Maminska A, Miaczynska M (2016). Endocytic regulation of cytokine receptor signaling. Cytokine Growth Factor. Rev..

[33] Akazawa Y (2009). Death receptor 5 internalization is required for lysosomal permeabilization by TRAIL in malignant liver cell lines. Gastroenterology.

[34] Kohlhaas SL, Craxton A, Sun XM, Pinkoski MJ, Cohen GM (2007). Receptor-mediated endocytosis is not required for tumor necrosis factor-related apoptosis-inducing ligand (TRAIL)-induced apoptosis. J. Biol. Chem..

[35] Aits S, Jaattela M (2013). Lysosomal cell death at a glance. J. Cell Sci..

[36] Werneburg NW, Guicciardi ME, Bronk SF, Kaufmann SH, Gores GJ (2007). Tumor necrosis factor-related apoptosis-inducing ligand activates a lysosomal pathway of apoptosis that is regulated by Bcl-2 proteins. J. Biol. Chem..

[37] Guicciardi ME, Bronk SF, Werneburg NW, Gores GJ (2007). cFLIPL prevents TRAIL-induced apoptosis of hepatocellular carcinoma cells by inhibiting the lysosomal pathway of apoptosis. Am. J. Physiol. Gastrointest. Liver Physiol.

[38] Hagiwara C, Tanaka M, Kudo H (2002). Increase in colorectal epithelial apoptotic cells in patients with ulcerative colitis ultimately requiring surgery. J. Gastroenterol. Hepatol..

[39] Zeissig S (2004). Downregulation of epithelial apoptosis and barrier repair in active Crohn’s disease by tumour necrosis factor alpha antibody treatment. Gut.

[40] Becker C, Watson AJ, Neurath MF (2013). Complex roles of caspases in the pathogenesis of inflammatory bowel disease. Gastroenterology.

[41] Gunther C (2011). Caspase-8 regulates TNF-alpha-induced epithelial necroptosis and terminal ileitis. Nature.

[42] Berg DJ (2002). Rapid development of colitis in NSAID-treated IL-10-deficient mice. Gastroenterology.

[43] Bertolotti A (2001). Increased sensitivity to dextran sodium sulfate colitis in IRE1beta-deficient mice. J. Clin. Invest..

[44] Hino K, Saito A, Asada R, Kanemoto S, Imaizumi K (2014). Increased susceptibility to dextran sulfate sodium-induced colitis in the endoplasmic reticulum stress transducer OASIS deficient mice. PLoS One.

