# Supplementary material for: Inflammatory bowel disease-associated ubiquitin ligase RNF183 promotes lysosomal degradation of DR5 and TRAIL-induced caspase activation
Source: Sci Rep. 2019 Dec 30;9:20301. doi: 10.1038/s41598-019-56748-6 (PMC6937276; doi:10.1038/s41598-019-56748-6)
Supplement: Supplementary file 1 — Supplementary information. [file 41598_2019_56748_MOESM1_ESM.pdf]

SUPPLEMENTARY INFORMATION

Inflammatory bowel disease-associated ubiquitin ligase RNF183 promotes lysosomal degradation of DR5 and TRAIL-induced caspase activation

Yan Wu[1], Yuka Kimura[1],Takumi Okamoto[1], Koji Matsuhisa[1], Rie Asada[2], Atsushi Saito[3], Fumika Sakaue[3], Kazunori Imaizumi[1,*], Masayuki Kaneko[1,*]

[1]Department of Biochemistry, Graduate school of Biomedical and Health Sciences, Hiroshima University, Hiroshima, Japan
[2]Department of Medicine, Division of Endocrinology, Metabolism, and Lipid Research, Washington University School of Medicine, MO, USA
[3]Department of Stress Protein Processing, Graduate School of Biomedical and Health Sciences, Hiroshima University, Hiroshima, Japan

[*]**Corresponding Authors**

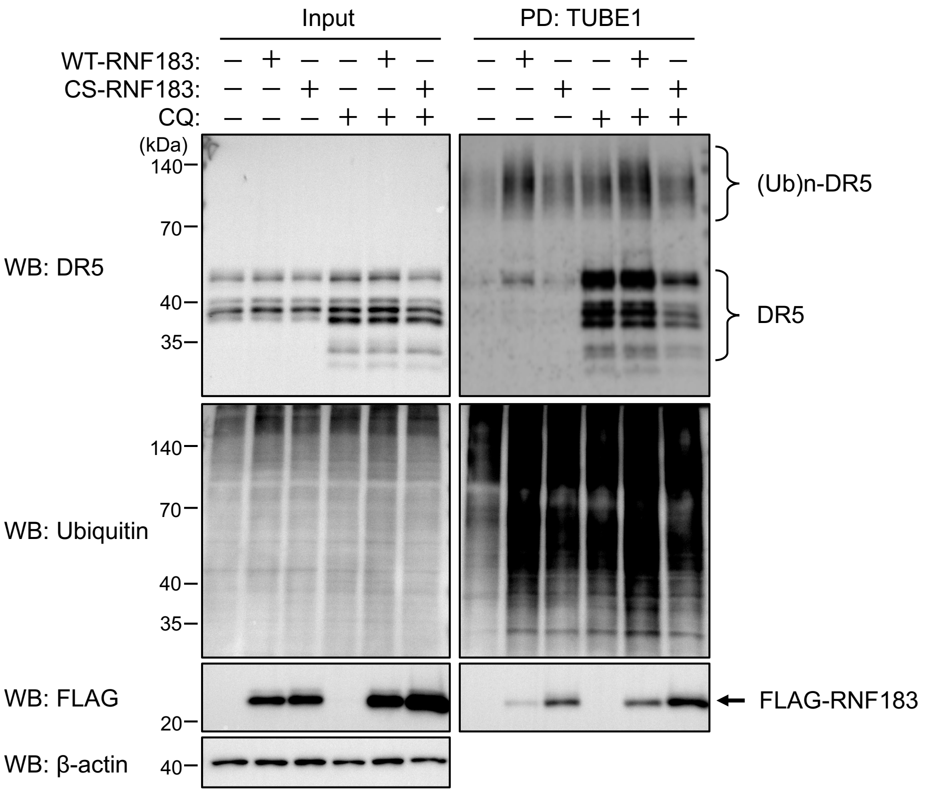

Supplementary Fig. S1. Tandem Ubiquitin Binding Entity (TUBE) assay for DR5 poly-ubiquitination by RNF183. We established clonal tetracycline-regulated HEK293 cell lines that inducibly express either wild-type (WT) or the C13S/C16S mutant of RNF183. Inducible HEK293 cells were treated with 1 μg/ml of doxycycline for 48 h. At 24 h before harvest, 100 μM chloroquine (CQ) was added. Cell lysates were treated with 15 μl of Agarose-TUBE1 (catalog no. 307406, LifeSensors, Malvern, PA) for 4 h at 4°C. Agarose-TUBE beads were washed with wash buffer and boiled with Laemmli sample buffer. The elutions were analyzed by western blotting with anti-DR5 or anti-ubiquitin (P4D1, mouse monoclonal; #3936, Cell Signaling Technology) antibodies (Pull-down, PD). The full-length blots are presented in Supplementary Fig. S13.

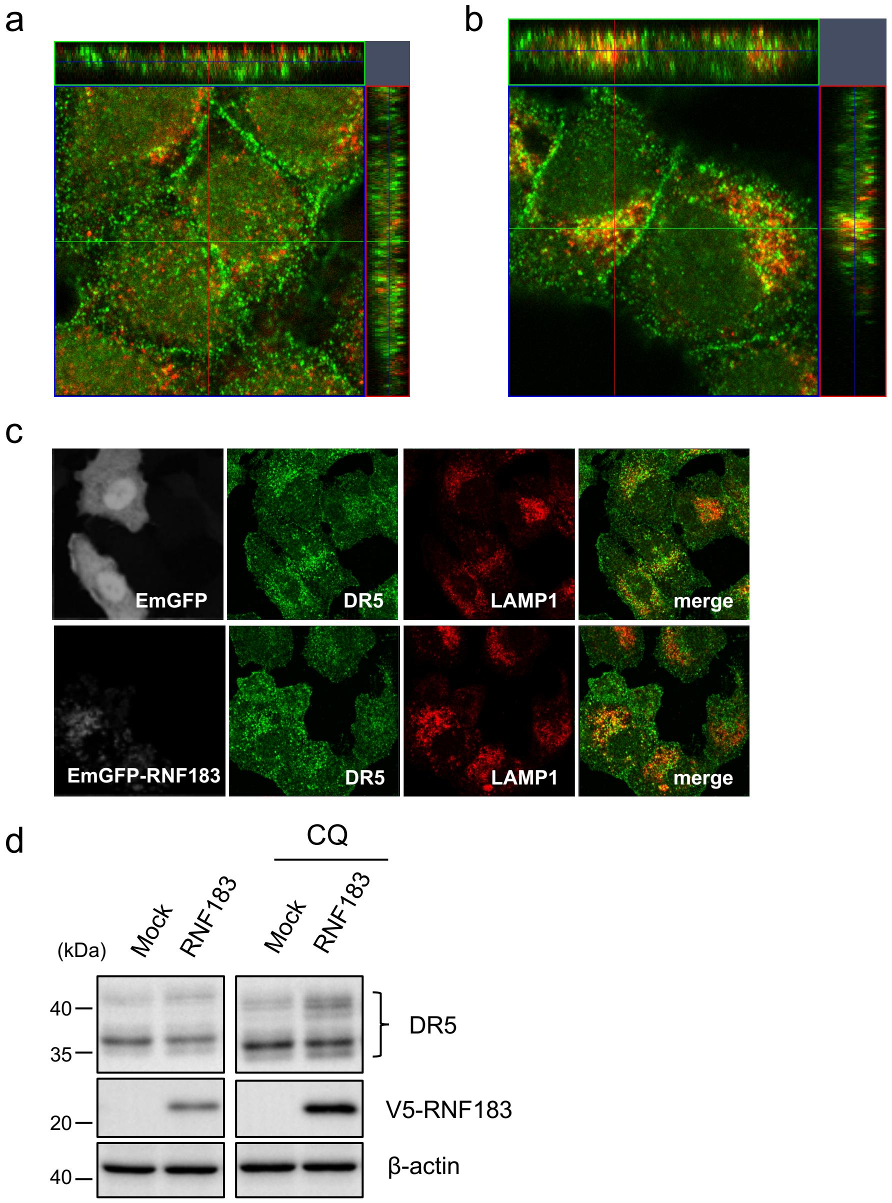

Supplementary Fig. S2. Lysosomal localization and degradation of DR5. (a, b) Confocal image stacks of Fig. 5a with (a) control EmGFP and (b) EmGFP-RNF183. (c) Effect of chloroquine on DR5 localization. HeLa cells transfected with EmGFP-tagged RNF183 and treated with 100 μM CQ for 12 h (green, DR5; red, LAMP1; gray, EmGFP). (d) Effect of chloroquine on DR5 protein levels. HEK293 cells expressing mock or V5-tagged RNF183 were treated with CQ for 12 h. Cell lysates were subjected to western blotting with anti-DR5 and anti-V5 antibodies. The full-length blots are presented in Supplementary Fig. S14.

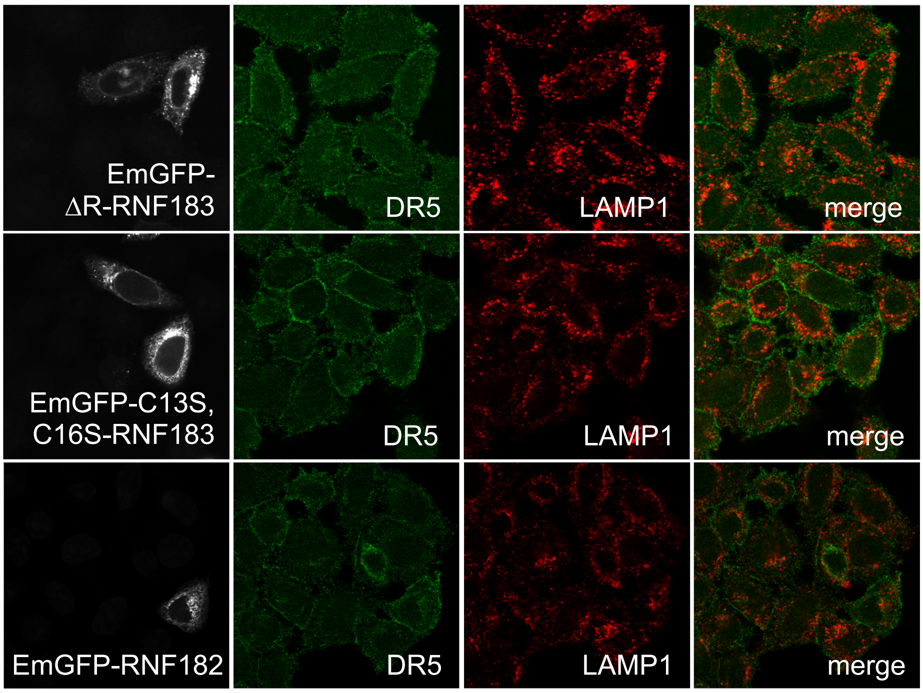

Supplementary Fig. S3. Effect of RNF183 mutants and RNF182 overexpression on DR5 subcellular localization. HeLa cells transfected with EmGFP-tagged RNF183 mutants (ΔRING-RNF183; C13S, C16S-RNF183) or RNF182 were subjected to immunofluorescence staining with DR5 and LAMP1 antibodies (*gray*, EmGFP; *green*, DR5; *red*, LAMP1).

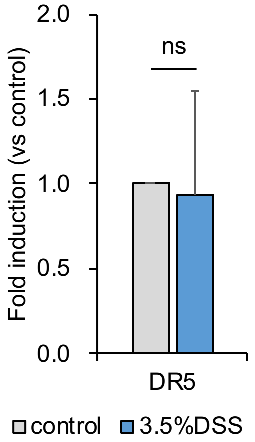

Supplementary Fig. S4. Expression of DR5 mRNA in isolated colonic epithelial cells of DW- and DSS-treated mice at 5 days. The expression levels were determined by qRT-PCR (n = 6; paired *t*-test; ns, no significant difference).

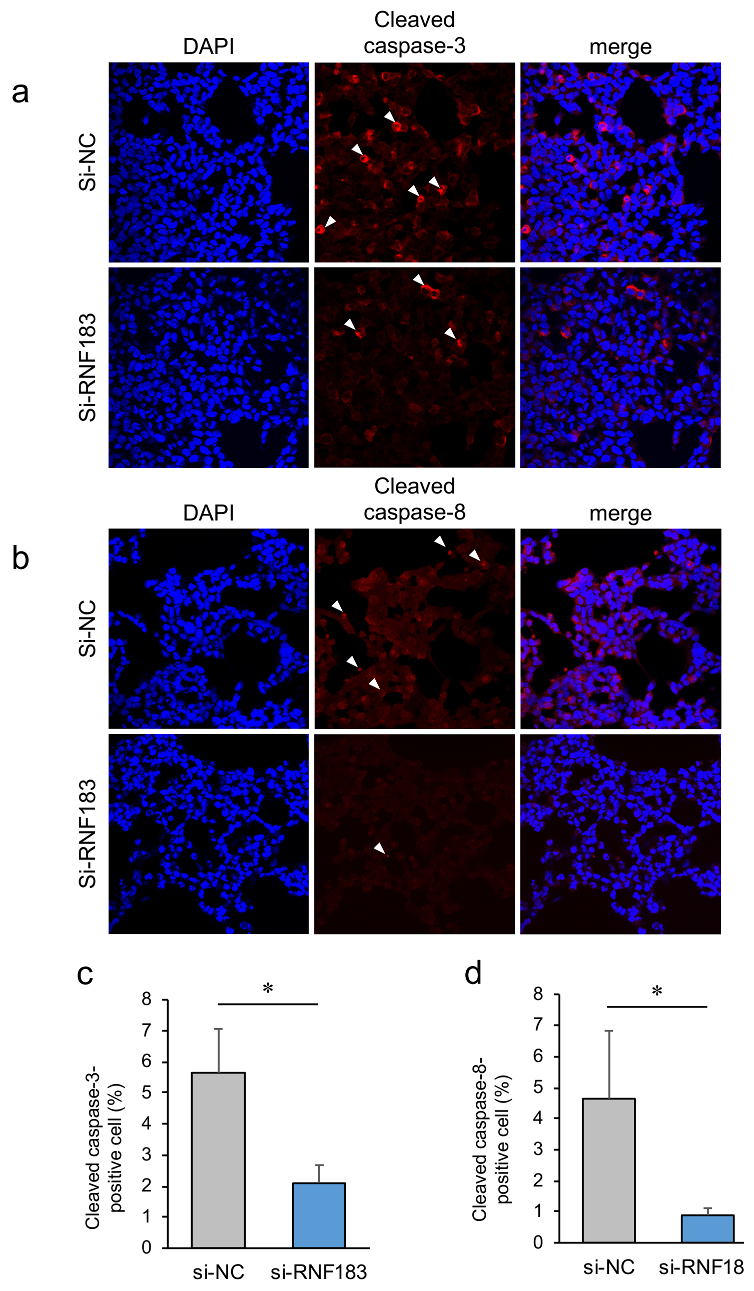

Supplementary Fig. S5. Effect of RNF183 knockdown on TRAIL-induced caspase activation. RNF183-siRNA was transfected into HEK293 cells stably expressing V5-tagged RNF183. At 24 h after transfection, TRAIL (100 ng/mL) was added and cells were incubated for 5 h. (a, b) Cells were subjected to immunofluorescence staining with cleaved caspase-3, cleaved caspase-8 antibodies (red), and DAPI (blue). (c, d) Quantitative graph of data from (a, b). Asterisks represent significant differences (n = 3; Student's t-test, *$p < 0.05$).

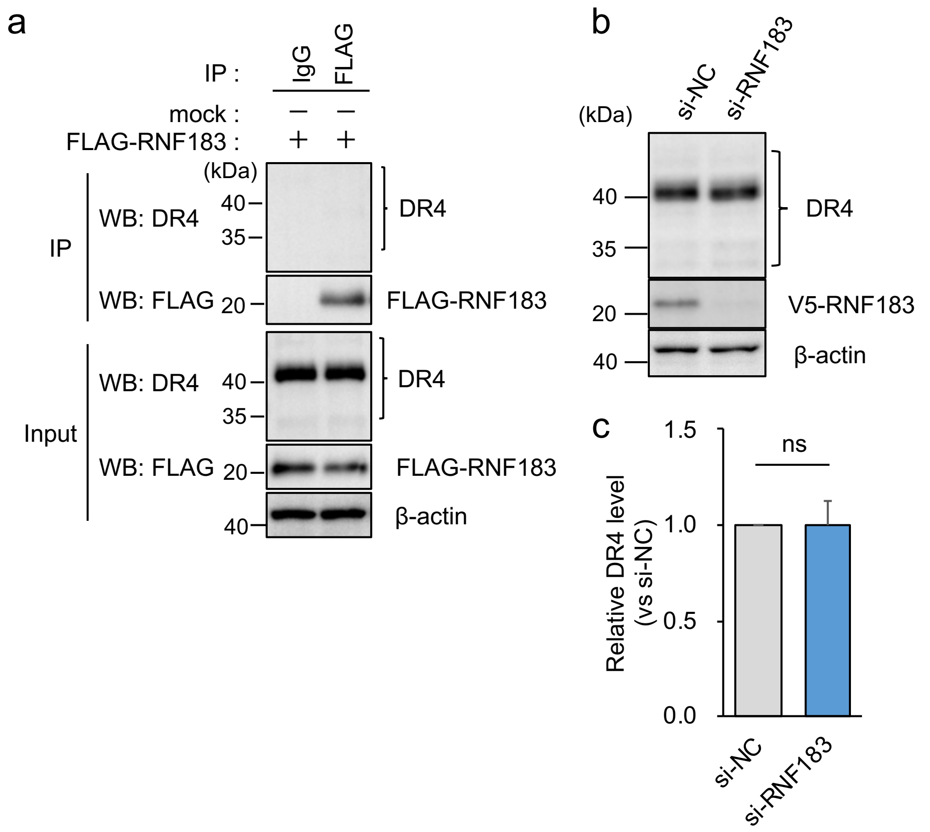

Supplementary Fig. S6. RNF183 is not associated with DR4. (a) Coimmunoprecipitation of RNF183 and DR4. Cell lysates from HEK293 cells stably expressing FLAG-tagged RNF183 were immunoprecipitated with anti-FLAG antibody, and the immune complexes were analyzed by western blotting with anti-DR4 or anti-FLAG antibodies. IP; immunoprecipitation. Full-length blots are presented in Supplementary Fig. S15. (b) Effect of RNF183 knockdown on DR4 protein levels. RNF183-siRNA or non-target control (NC)-siRNA was transfected into HEK293 cells stably expressing V5-tagged RNF183. Cell lysates were subjected to western blotting with anti-DR4 and anti-V5 antibodies. Full-length blots are presented in Supplementary Fig. S16. (c) Quantitative graph of data from (b). n = 4; paired *t*-test, ns: no significant difference.

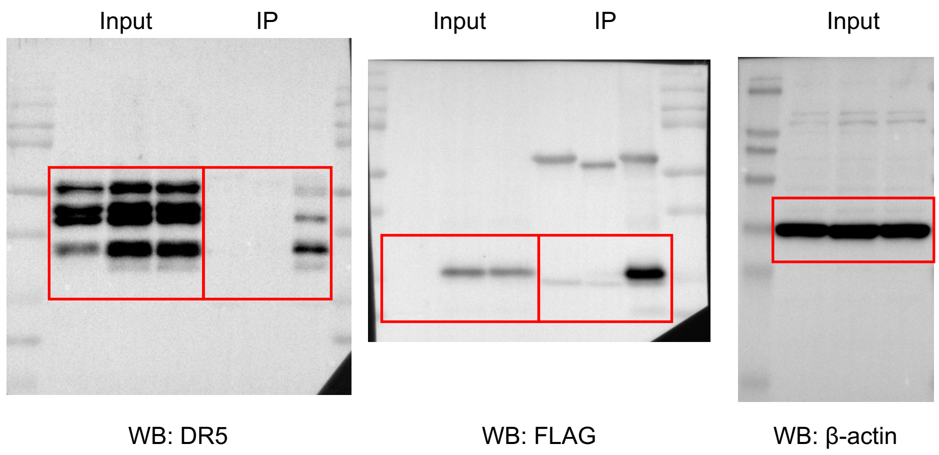

Supplementary Fig. S7. Full-length Western blot images for Fig. 4a.

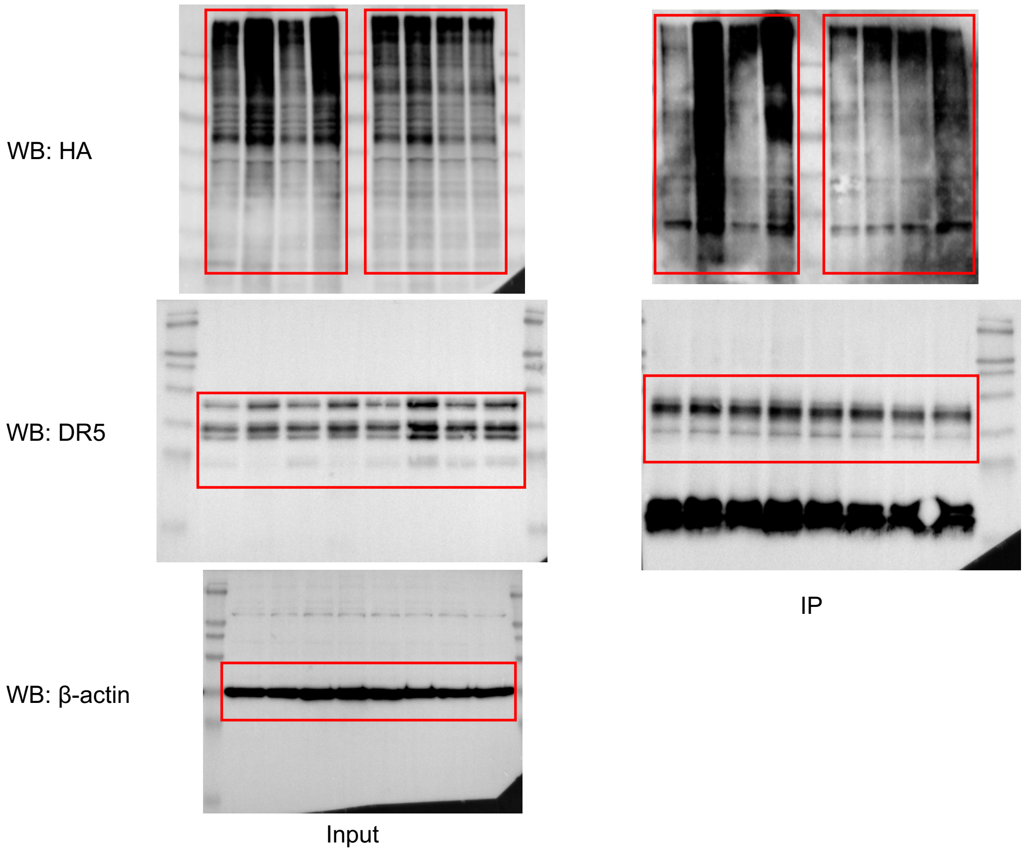

Supplementary Fig. S8. Full-length Western blot images for Fig. 4b.

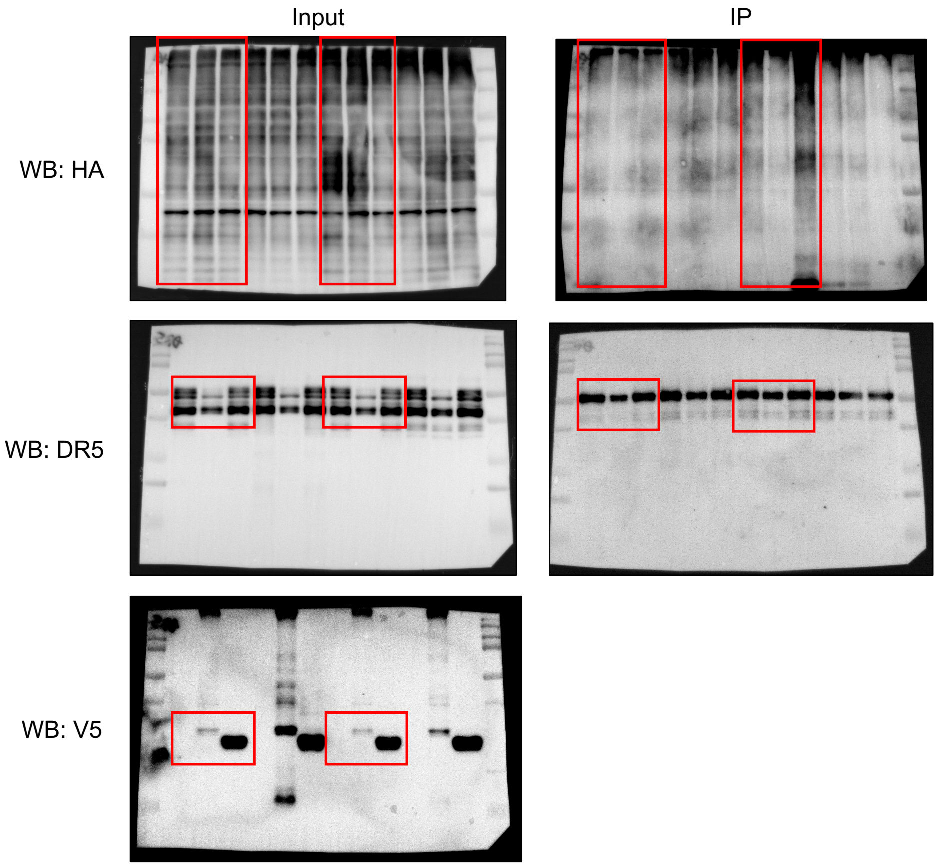

Supplementary Fig. S9. Full-length Western blot images for Fig. 4d.

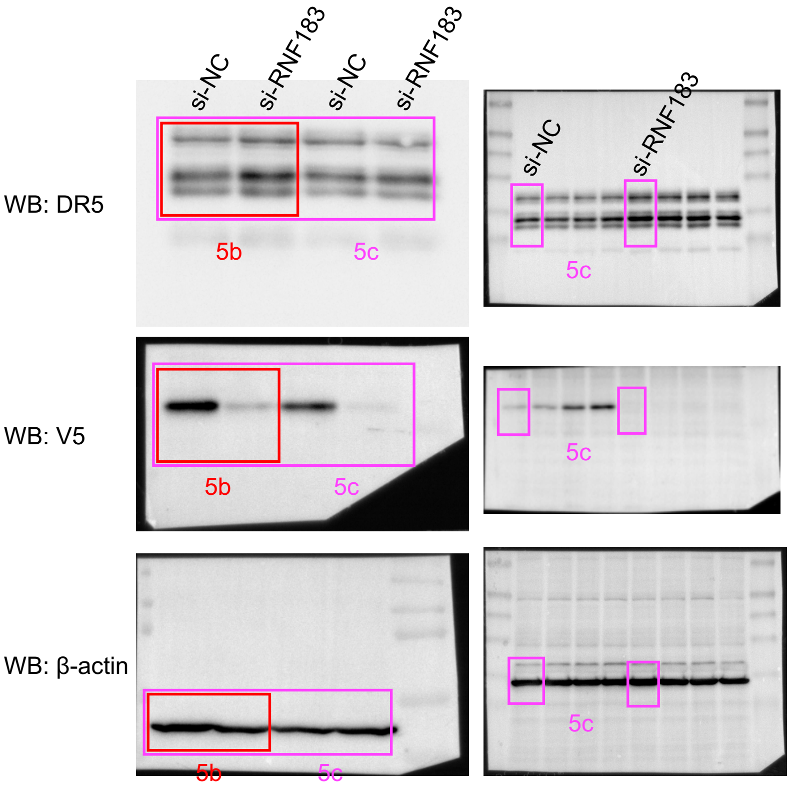

Supplementary Fig. S10. Full-length Western blot images for Fig. 5b and c.

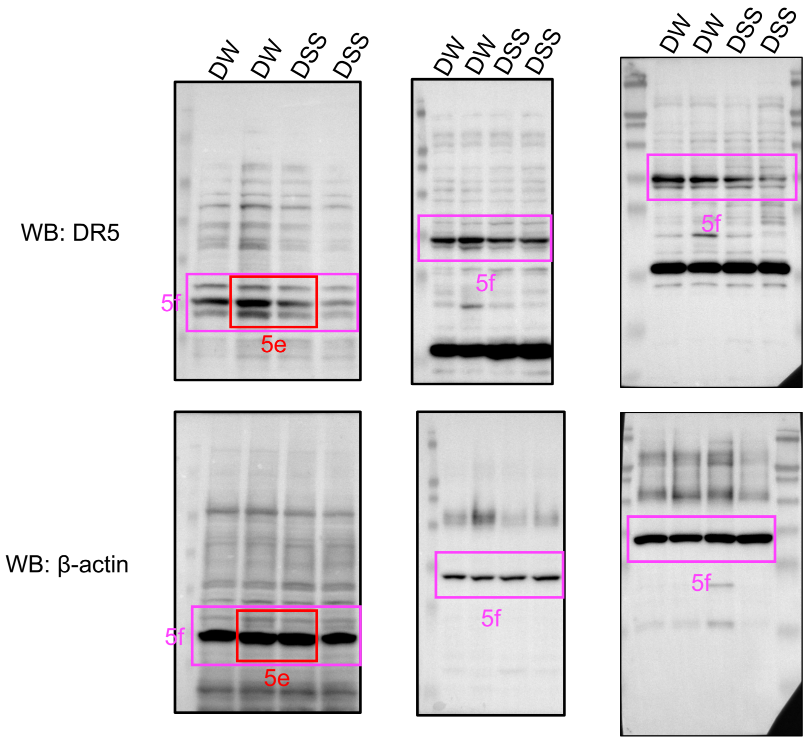

Supplementary Fig. S11. Full-length Western blot images for Fig. 5e and f.

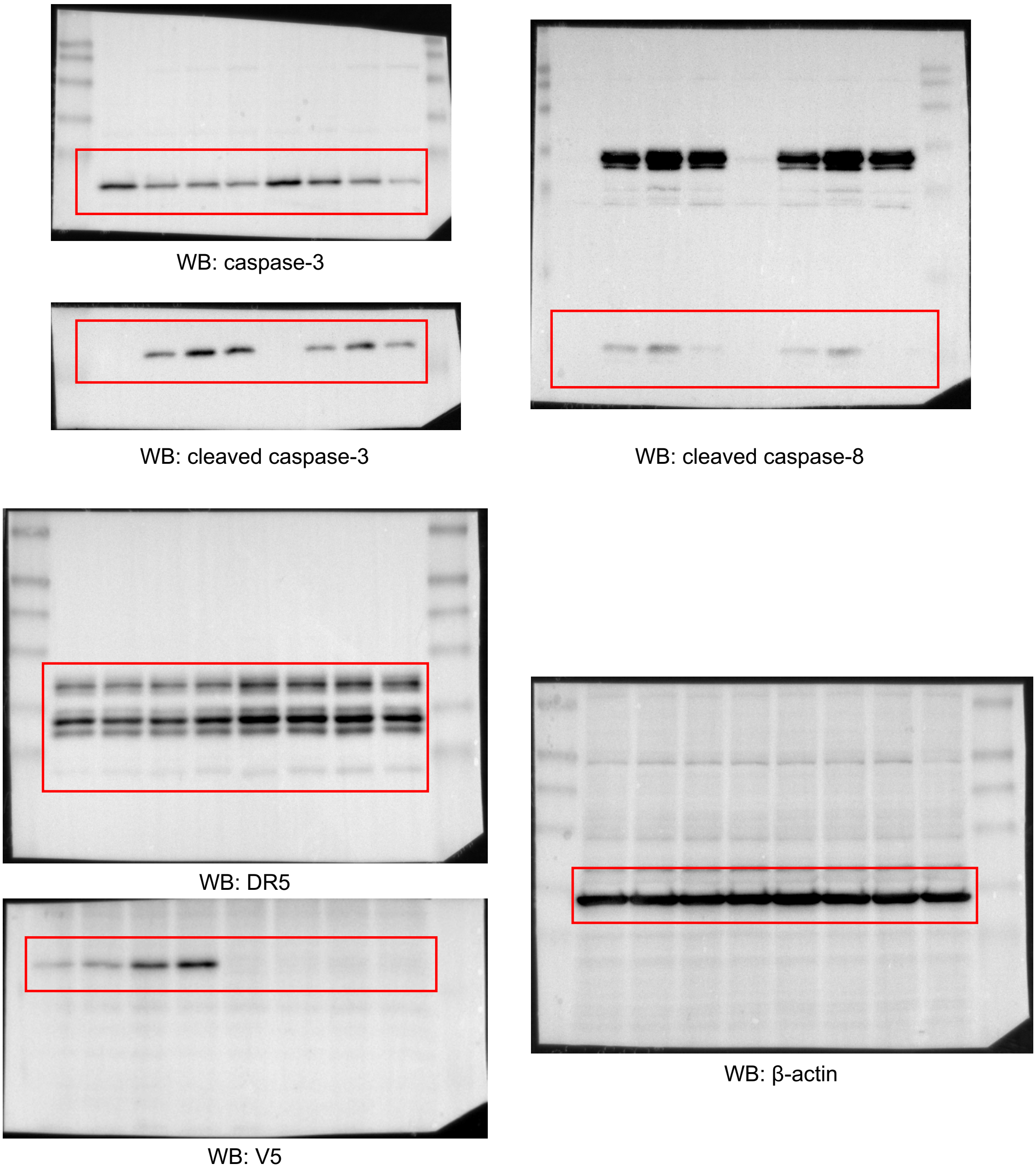

Supplementary Fig. S12. Full-length Western blot images for Fig. 6a.

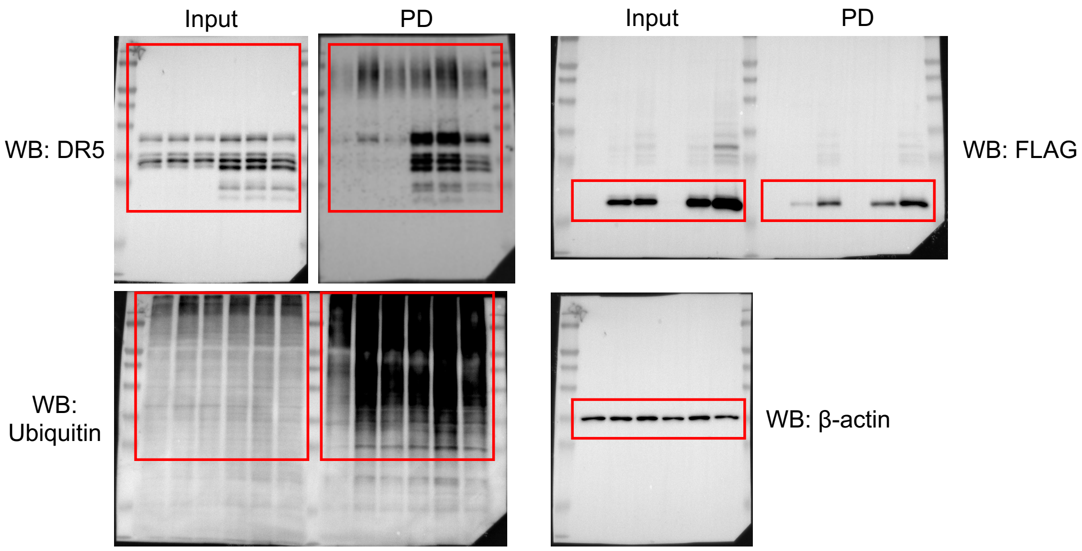

Supplementary Fig. S13. Full-length Western blot images for Supplementary Fig. S1.

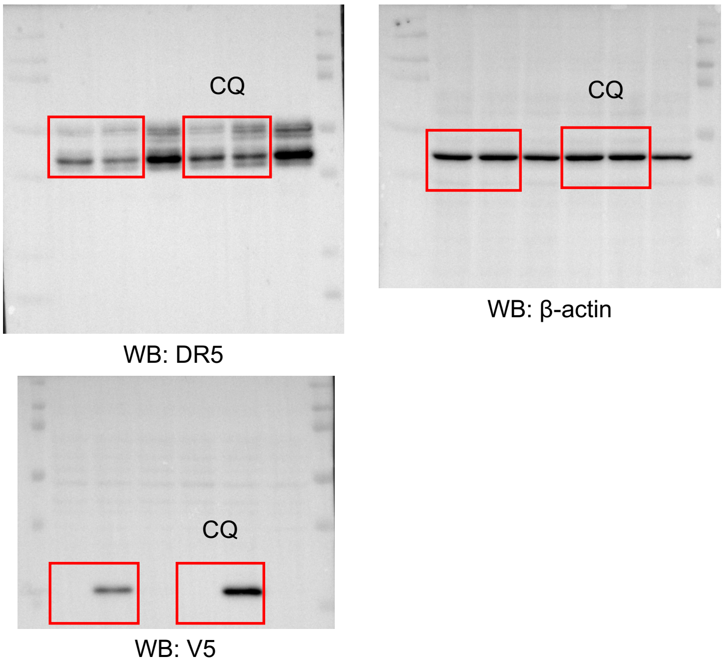

Supplementary Fig. S14. Full-length Western blot images for Supplementary Fig. S2d.

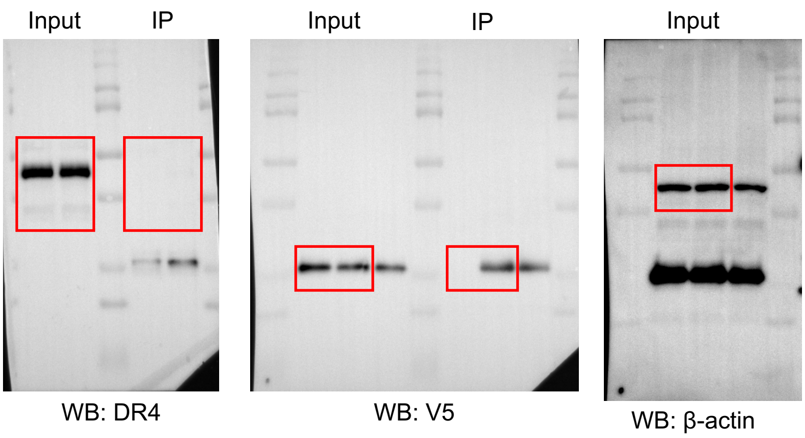

Supplementary Fig. S15. Full-length Western blot images for Supplementary Fig. S6a.

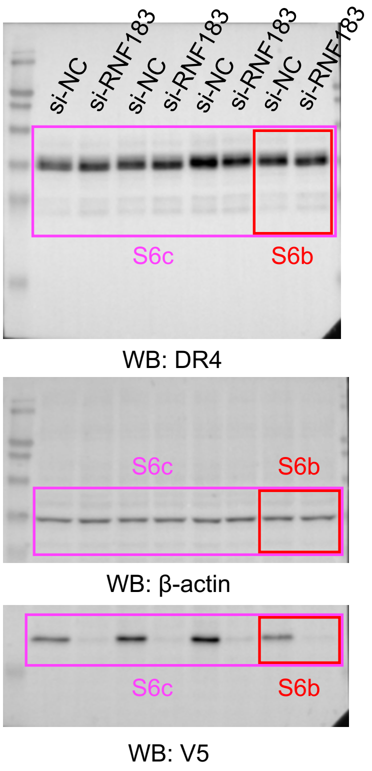

Supplementary Fig. S16. Full-length Western blot images for Supplementary Fig. S6b and c.
